# Research Progress on the Anti-Inflammatory and Antioxidant Effects of Daidzein: Its Mechanisms of Action in Related Diseases, and Related Nanoformulations to Enhance Its Bioavailability

**DOI:** 10.3390/antiox15060775

**Published:** 2026-06-22

**Authors:** Xinxin Chen, Han Di, Gang Wang, Yanhong Wang, Feng Guan

**Affiliations:** 1 School of Pharmacy, Heilongjiang University of Chinese Medicine, Harbin 150040, China; 2 Key Laboratory of Basic and Application Research of Beiyao, Ministry of Education, Heilongjiang University of Chinese Medicine, Harbin 150040, China

**Keywords:** daidzein, phytoestrogens, anti-inflammatory, antioxidant, oxidative stress, nanodelivery systems

## Abstract

Daidzein is a naturally occurring isoflavone phytoestrogen, mainly found in leguminous plants. This component exerts anti-inflammatory effects by regulating inflammatory cells via multiple targets, blocking core inflammatory pathways, and inhibiting the release of inflammatory factors. It also scavenges reactive oxygen species, activates the antioxidant enzyme system, and regulates antioxidant signaling pathways to achieve antioxidant effects. By regulating these two core pathological processes, it exerts protective effects in diseases such as cancer, cardiovascular disease, and acute kidney injury, based on preclinical evidence. The development of nanodelivery systems has effectively improved the physicochemical properties of daidzein, enhanced its bioavailability, and enabled disease-targeted delivery. Most previous reviews have either focused exclusively on daidzein or broadly covered the pharmacological activities of isoflavones, yet have largely overlooked the dual anti-inflammatory and antioxidant mechanisms specific to daidzein. This review summarizes these mechanisms and their preclinical effects on various diseases, including cancer, cardiovascular diseases, and acute kidney injury. It also reviews the pharmacokinetic limitations of daidzein and recent progress in nanodelivery strategies aimed at enhancing its bioavailability and bioactivity. Overall, this review serves as a reference for the future standardized comparison of nanocarriers, targeted therapies, and clinical applications.

## 1. Introduction

Oxidative stress and chronic inflammation are two closely related core pathological processes that are central to the occurrence and development of various human diseases. They mutually reinforce each other, forming a vicious cycle that exacerbates cellular damage and tissue dysfunction [1]. Excessive reactive oxygen species (ROS) generation triggered by oxidative stress activates inflammatory signaling pathways, and the ensuing inflammatory response further induces ROS production. Inflammation and oxidative stress, as interwoven core pathological processes, cause cell damage, dysfunction, and even tissue fibrosis by regulating key signaling pathways such as nuclear factor kappa-B (NF-κB), mitogen-activated protein kinase (MAPK), and nuclear factor erythroid 2-related factor 2 (Nrf2) [2]. Inflammation and oxidative stress are also involved in the pathological mechanisms of major diseases such as cancer, cardiovascular disease (CVD), and acute kidney injury (AKI) [3]. In recent years, natural plant-derived active ingredients have received extensive attention in the field of disease prevention and treatment due to their advantages such as multi-targeted regulation, low toxicity, and few side effects, thus they have become important research subjects for the development of novel therapeutic drugs and functional food additives [4]. Phytoestrogens are a class of natural compounds widely present in plants that possess estrogen-like activity. They exert significant regulatory effects on oxidative stress and inflammatory responses, and their potential therapeutic value in various chronic diseases is increasingly recognized [5].

Daidzein is a typical isoflavone phytoestrogen and a characteristic active constituent of leguminous plants [6]. It possesses multiple biological activities, including anti-inflammatory, antioxidant, and metabolic regulatory effects, and has low toxicity and wide availability, thus attracting considerable attention from the academic community [7]. Daidzein’s unique C6-C3-C6 carbon skeleton and hydroxyl functional groups at specific positions confer high affinity and diverse biological activities with estrogen receptor β (ERβ) [8]. As a common dietary active ingredient, daidzein is mainly consumed by humans from soybeans and their products. Due to differences in dietary structure, the intake in the Asian population can reach 50 mg/d, which is much higher than that in the Western population [9]. In recent years, numerous in vivo and in vitro studies have confirmed that daidzein can precisely regulate the functions of inflammatory cells, block core inflammatory pathways such as NF-κB and MAPK, and simultaneously activate antioxidant signaling pathways such as the Nrf2/HO-1 axis, thereby exerting a wide range of pharmacological effects, including antioxidant [10], anti-inflammatory [11], and anti-tumor effects [12]. However, daidzein is plagued by inherent pharmacokinetic deficiencies, such as poor aqueous solubility, low oral bioavailability, and dependence on intestinal enzymatic activation, thereby greatly hindering its clinical translation and industrial application [13]. To overcome these inherent pharmacokinetic bottlenecks of natural active ingredients, nanodelivery systems have emerged as a promising strategy for practical application [14]. Accordingly, researchers have developed a variety of nanodelivery systems through formulation optimization, effectively enhancing their solubility, stability, and targeted delivery efficiency, and significantly boosting their biological activity [15,16].

This review summarizes the structural origin of daidzein, its anti-inflammatory and antioxidant molecular mechanisms, its regulatory effects on related diseases, as well as its pharmacokinetic limitations and recent advances in nanodelivery systems. Together, these aspects provide a scientific foundation for future research on daidzein and offer new insights into the development of novel natural drugs targeting diseases associated with oxidative stress and inflammation.

## 2. Methodology

This review encompasses relevant literature retrieved from databases including PubMed, Web of Science, and Google Scholar using keywords such as daidzein, antioxidants, anti-inflammatory effects, and nanodelivery systems, and covering mainly articles published in the past two decades. Conference abstracts, patents, and review articles lacking original data were excluded. The review summarizes the anti-inflammatory and antioxidant properties of daidzein, its effects in diseases including cancer, cardiovascular diseases, and acute kidney injury, as well as the improved bioavailability and related activities of its nanoformulations.

## 3. Chemical Structure and Main Sources of Daidzein

### 3.1. Chemical Structure and Chemical Information

Daidzein, also known as 7,4′-dihydroxyisoflavone, is a naturally occurring plant estrogen belonging to the non-steroidal estrogen category [17]. It has a basic carbon skeleton of C6-C3-C6, with a molecular formula of C_15_H_10_O_4_ and a molecular weight of 254.24. Pure daidzein is a pale yellow crystal with a melting point in the range of 315–323 °C. It is insoluble in water but readily soluble in organic solvents such as methanol and ethanol. Due to its poor water solubility, the oral bioavailability of daidzein is only 20–30% [18]. This figure is calculated as the ratio of total plasma isoflavones (both bound and free forms) to the intake dose. The data are mostly derived from studies on pure aglycone preparations or specific processed foods [19]. The hydroxyl groups at C7 of ring A and position 4′ of ring B are the core functional groups, which enable daidzein to preferentially bind to ERβ and confer its phytoestrogenic activity [20].

**Figure 1:**
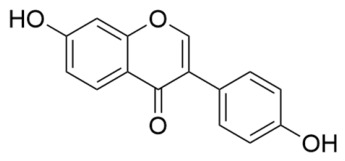
Chemical structure of daidzein.

In leguminous plants, daidzein predominantly presents in glycosylated forms. The glycosidic group needs to be hydrolyzed by intestinal enzymes to generate free aglycone, which is responsible for the biological and physiological functions of daidzein [21]. In plants, daidzein is synthesized via the phenylpropanoid pathway, and its biosynthesis is tightly regulated by specific enzymes, cofactors, and related regulatory genes. The phenolic hydroxyl groups of daidzein are susceptible to chemical modifications, including esterification, sulfonation, and methylation. Certain daidzein derivatives possess stronger biological activities than the parent molecule. Moreover, thermal treatment and fermentation can modify its chemical form and abundance, thus elevating the ratio of free aglycones [22]. Its structure is shown in Figure 1.

### 3.2. Primary Sources

Daidzein is a characteristic isoflavone component of leguminous plants, with natural sources including *Glycine max* (L.) Merr., *Trifolium pratense* L., *Medicago sativa* L., and *Pueraria lobata* [18]. Soybeans are the primary and richest natural dietary source of daidzein. However, the reported content of daidzein varies significantly, mainly influenced by factors such as the variety, processing methods, fermentation process, and analytical methods [23].

Specifically, the content of soybean glycosides can vary by several times among different varieties [24]. In terms of processing, heat treatment can cause some soybean glycosides to dissolve or degrade [25]. During the fermentation process, with the help of microbial β-glucosidase, the glycosides are converted into more active aglycone forms, thereby increasing the bioavailability of daidzein [26]. Additionally, differences in analytical methods and sample pretreatment procedures can also lead to variations in quantitative results [27].

When daidzein is consumed as a daily dietary component, it is mainly obtained from beans and their sprouted products, with a daily dosage ranging from a few to tens of milligrams. It helps maintain health over the long-term but does not produce acute pharmacological effects [28]. When used as a medicinal component, it involves the use of purified extracts or dietary supplements at high doses of more than 50 mg per day for specific therapeutic purposes such as alleviating menopausal symptoms and promoting bone health. However, high doses may cause side effects, including reproductive toxicity [26].

Traditionally, daidzein was considered present only in leguminous vegetables and grains [29,30]. However, recent studies have detected daidzein in non-leguminous plants such as *Iris* L., *Chenopodium* L., and *Pistacia* L. [31,32]. These findings have challenged the traditional perception that its source is mainly restricted to the legume family. Nevertheless, studies have also shown that the content of daidzein in these non-leguminous plants is much lower than that in leguminous plants. Currently, related fermented products and dietary supplements on the market still use leguminous soybeans as the core ingredient [33]. The main sources of daidzein are summarized in Table 1.

## 4. Anti-Inflammatory and Antioxidant Mechanisms of Daidzein

### 4.1. Anti-Inflammatory Mechanism

**Figure 2:**
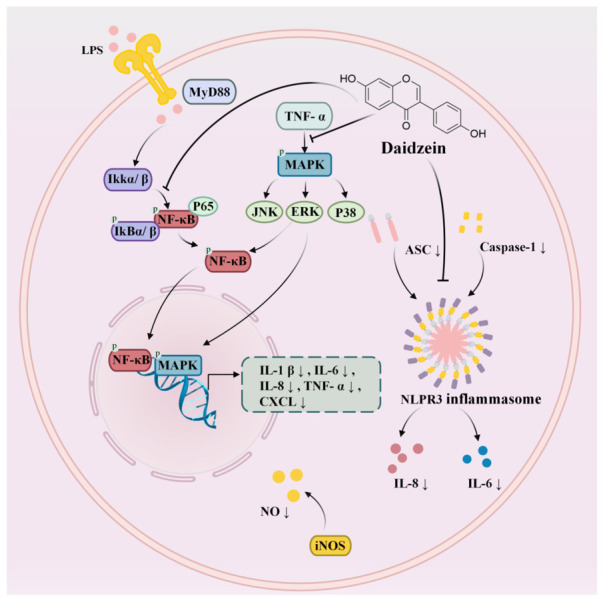
Mechanisms involved in the anti-inflammatory effects of daidzein. Note: ASC, apoptosis-associated speck-like protein containing a CARD; caspase-1, cysteine-aspartic acid protease 1; CXCL, C-X-C motif chemokine ligand; ERK, extracellular signal-regulated kinase; IkBα/β, inhibitor of kappa B α/β; IKKα/β, inhibitor of kappa B kinase α/β; iNOS, inducible nitric oxide synthase; IL-1β, interleukin-1β; IL-6, interleukin-6; IL-8, interleukin-8; JNK, c-Jun N-terminal kinase; LPS, lipopolysaccharide; MAPK, mitogen-activated protein kinase; MyD88, myeloid differentiation primary response 88; NF-κB P65, nuclear factor kappa-light-chain-enhancer of activated B cells p65 subunit; NLPR3, NOD-like receptor thermal protein domain associated protein 3; NO, nitric oxide; TNF-α, tumor necrosis factor-α.

Daidzein regulates the initiation, amplification, and persistence of the inflammatory response via multiple targets and mechanisms. The core mechanism involves the regulation of inflammatory cell functions and the blockade of key signaling pathways. The anti-inflammatory mechanism of daidzein is illustrated in Figure 2.

#### 4.1.1. Daidzein Regulates the Function of Inflammatory Cells

Daidzein can effectively inhibit the polarization of macrophages toward a pro-inflammatory phenotype and reprogram them to an anti-inflammatory state. The core mechanism lies in the regulation of the NOD-like receptor thermal protein domain associated protein 3 (NLRP3) inflammasome signaling pathway. In RAW264.7 cells induced by lipopolysaccharide (LPS) at 1 μg/mL, treatment with 10–40 μmol/L daidzein for 48 h in the presence of high glucose (30 mmol/L) or additional LPS significantly downregulated the expression of the M1 polarization markers inducible nitric oxide synthase (iNOS), cluster of differentiation 80 (CD80), and cluster of differentiation 86 (CD86) [43]. It also significantly reduced the protein expression levels of Toll-like receptor 4 (TLR4) and myeloid differentiation primary response 88 (MyD88) in macrophages, thereby inhibiting the activation of downstream NF-κB. Additionally, daidzein downregulated the expression of NLRP3 inflammasome, ASC, and pro-caspase-1, as well as the production of ROS. Another study using 10–100 μmol/L daidzein or its aglycone to pre-treat cells for 4 h, followed by LPS stimulation for 12 h (with 5 μmol/L dexamethasone as a positive control), confirmed the regulatory effect on this pathway [44]. Through these mechanisms, daidzein ultimately reduced the secretion of tumor necrosis factor-α (TNF-α), interleukin-1β (IL-1β), and interleukin-18 (IL-18), and alleviated macrophage-mediated inflammatory damage.

Daidzein can effectively inhibit the maturation and function of dendritic cells (DCs), thereby exerting immunosuppressive effects at the interface between innate and adaptive immunity. A study using bone marrow-derived DCs from C57BL/6 mice as a model found that treatment with 1–20 μmol/L daidzein in the presence of 10 ng/mL lipopolysaccharide (LPS) for 20 h significantly suppressed the expression of multiple maturation markers on DCs, including cluster of differentiation 40 (CD40), CD80, CD86, and major histocompatibility complex class II (MHC class II) molecules. Additionally, after 4 h of treatment, daidzein markedly reduced the ability of DCs to produce IL-12p40, interleukin-6 (IL-6), and TNF-α while restoring the endocytic activity of DCs and attenuating their ability to stimulate the proliferation of allogeneic CD4^+^ T cells [45].

Daidzein can regulate microglial cells to alleviate inflammatory damage. In LPS-induced BV2 microglial cell models, pretreatment with 10–50 μmol/L daidzein for 12 h before stimulation with 1 μg/mL LPS significantly reduced the production of pro-inflammatory mediators such as nitric oxide (NO), prostaglandin E2 (PGE2), IL-6, and IL-1β [46]. By inhibiting microglial activation, daidzein reduces oxidative stress and the release of inflammatory factors, thereby blocking the neuroinflammatory cascade and exerting an indirect neuroprotective effect that alleviates neuroinflammatory damage.

#### 4.1.2. Daidzein Blocks the Core Inflammatory Signaling Pathways

NF-κB is the core transcription factor of the inflammatory response, and daidzein exerts regulatory effects at multiple key levels of the NF-κB pathway, thereby inhibiting the release of inflammatory mediators, regulating immune cell activation, and exerting broad anti-inflammatory activity. Daidzein significantly inhibits IKKα/β phosphorylation and blocks the upstream activation signal of the NF-κB pathway [47]. By inhibiting inhibitor of kappa B kinase (IKK) activity, it prevents the phosphorylation and degradation of IκBα, allowing IκBα to continuously bind and inhibit NF-κB [44]. Meanwhile, daidzein suppresses p65 phosphorylation and nuclear translocation [48], which further inhibits NF-κB transcriptional activity and downregulates downstream inflammatory gene expression. Moreover, it targets upstream TLR4/MyD88 and modulates the ERK1/2 and other MAPK signaling cascades, thereby synergistically strengthening anti-inflammatory responses [49,50].

Based on the hierarchical regulatory effects of the NF-κB pathway described above, daidzein can further downregulate the transcription, translation, and secretion of key pro-inflammatory factors and inflammatory mediators mediated by NF-κB, thereby blocking the amplification of the inflammatory cascade at its terminal stage. Studies have confirmed that daidzein can significantly inhibit the expression and release of core pro-inflammatory cytokines such as TNF-α, IL-1β, IL-6, and interleukin-8 (IL-8) [51,52]. These factors are key effector molecules that drive inflammatory responses and recruit immune cells following activation of the NF-κB pathway. Additionally, daidzein reduces the activity and expression of inflammation-related enzymes, including iNOS and cyclooxygenase-2 (COX-2), decreases the production of inflammatory mediators such as NO and PGE2, and alleviates inflammatory pathological manifestations such as local tissue redness, swelling, and tissue damage [53,54].

The MAPK pathway is another key inflammatory signaling pathway, and daidzein can simultaneously inhibit the activation of its three major branches. In an oral squamous cell carcinoma (OSCC) cell model, daidzein treatment significantly reduced the phosphorylation levels of p38 and ERK [55]. In mouse abdominal aortic aneurysm models, daidzein inhibits TNF-α and IL-1β by regulating pathways such as p38 and transforming growth factor-β (TGF-β1), and modulates the expression of related molecules including COX-2 and matrix metallopeptidase 2 (MMP-2), thereby reducing vascular inflammation and aneurysm dilation [56]. The activation of p38, ERK1/2 and JNK is closely related to the production of inflammatory factors and cellular stress responses. Daidzein inhibits the activation of these three pathways, which synergizes with the inhibition of NF-κB to reduce the production of pro-inflammatory mediators and amplify the anti-inflammatory effect.

The activation of the Nrf2/HO-1 pathway represents a key mechanism underlying the anti-inflammatory effects of daidzein. Studies have shown that daidzein treatment significantly increases the protein levels of Nrf2 downstream target genes, including heme oxygenase-1 (HO-1) and NAD(P)H dehydrogenase quinone 1 (NQO1) [57]. HO-1 can break down pro-oxidative heme and produce metabolites with anti-inflammatory effects, whereas NQO1 detoxifies quinone substances and reduces oxidative damage. Daidzein upregulates Nrf2 expression and promotes its nuclear translocation, thereby upregulating the expression of downstream antioxidant proteins and enhancing antioxidant enzyme activity via the Akt/GSK-3β/Nrf2 signaling pathway [58]. By enhancing antioxidant defense, daidzein facilitates the clearance of ROS [59], thereby reducing oxidative stress-induced activation of inflammatory pathways at its source. This action complements and acts synergistically with the direct inhibition of NF-κB and MAPK, leading to more comprehensive control of inflammation.

Daidzein downregulates the expression of chemokines, thereby reducing local immune cell infiltration and inflammatory amplification. In TNF-α-induced mouse lung inflammation models, daidzein antagonizes TNF-α-induced Cxcl2 expression and activity, decreases NF-κB transcriptional activity, inhibits poly(ADP-ribose) polymerase-1 (PARP-1) enzyme activity, suppresses neutrophil infiltration into lung tissue, and alleviates local inflammation [60].

### 4.2. Antioxidant Mechanisms of Daidzein

**Figure 3:**
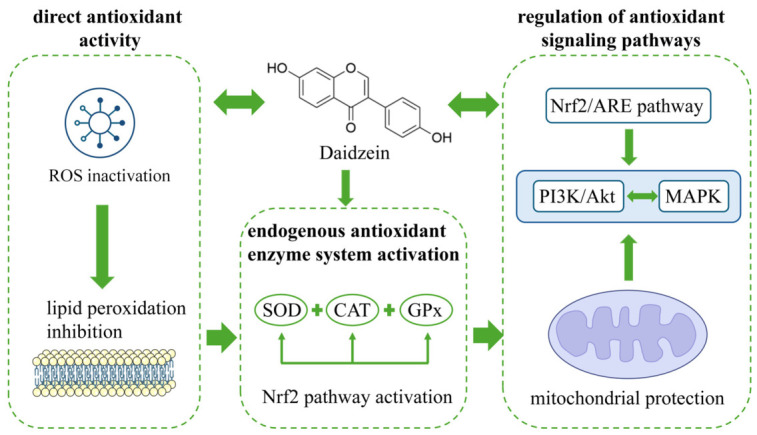
Antioxidant mechanism of daidzein. Note: CAT, catalase; GPx, glutathione peroxidase; MAPK, mitogen-activated protein kinase; Nrf2, nuclear factor erythroid 2-related factor 2; PI3K/Akt, phosphatidylinositol 3-kinase/protein kinase B; ROS, reactive oxygen species; SOD, superoxide dismutase.

Daidzein maintains oxidative balance via a triple mechanism, including direct ROS scavenging, activation of antioxidant enzyme systems, and regulation of signaling pathways, thereby protecting against ROS-mediated cellular damage. Recent studies have further demonstrated the enhanced bioactivity of its metabolites and their tissue-specific antioxidant properties. The antioxidant mechanism of daidzein is illustrated in Figure 3.

#### 4.2.1. ROS Inactivation

Daidzein promotes the scavenging of ROS by enhancing the endogenous antioxidant system. In the pig intestinal epithelial cell model [61], daidzein upregulates the expression of antioxidant enzymes such as SOD and CAT, thereby reducing the intracellular ROS level induced by H_2_O_2_ and decreasing the production of the lipid peroxidation product MDA. In the mouse testicular cell model [62], although ROS levels were not directly measured, daidzein significantly restored the SOD activity and GSH content inhibited by A1254, thereby enhancing the cells’ ability to remove superoxide anions; it also reduced the MDA level and alleviated cell damage.

However, it is critical to emphasize that daidzein exhibits bidirectional antioxidant/pro-oxidant activity, which is highly relevant for cancer biology and safety assessment. In multiple cancer cell models, daidzein increases ROS levels through pro-oxidation, regulates β-cell lymphoma 2 (Bcl-2) family proteins, activates related caspase proteases, and induces decreased mitochondrial membrane potential, DNA damage, and cell cycle arrest, ultimately leading to tumor cell apoptosis [63,64,65]. In normal cells, daidzein exerts antioxidant effects by directly scavenging ROS. In cancer cells, however, it functions as a pro-oxidant, selectively enhancing oxidative stress. This dual activity is context-dependent, varying with dosage, cell type, estrogen receptor status, and the microenvironment. From a safety perspective, this duality warrants caution, as high doses or specific conditions may trigger pro-oxidative toxicity.

#### 4.2.2. Daidzein Activates the Endogenous Antioxidant Enzyme System

Daidzein not only directly eliminates ROS but also enhances the long-term antioxidant defense of cells by upregulating the expression of endogenous antioxidant enzymes [66]. In various animal models and cell experiments, daidzein treatment has been shown to significantly increase the activity or expression levels of key antioxidant enzymes, thereby forming a multi-level enzymatic antioxidant network.

From one perspective, it activates antioxidant enzymes such as SOD, catalase (CAT), glutathione peroxidase (GPx), enhances the function of the glutathione system, and reduces oxidative damage products such as MDA. For example, in myocardial and spinal cord ischemia-reperfusion injury models, this compound upregulates the activities of SOD, CAT, and GPx and reduces MDA accumulation [67,68]. Moreover, it enhances GSH content and CAT activity in glycerol-induced AKI in a dose-dependent fashion [69].

In contrast, the Nrf2 pathway is activated in both porcine small intestinal epithelial cells and traumatic brain injury models, accompanied by the upregulated expression of phase II detoxifying enzymes including HO-1 and NQO1 [61,70]. These endogenous antioxidant enzymes act synergistically to markedly reduce cellular ROS levels and mitigate lipid peroxidation as well as oxidative damage to proteins and DNA. For example, daidzein protects antioxidant enzyme activity in human skin cells [71], enhances SOD and GSH activity, and reduces lipid peroxidation in glioblastoma experiments [72]. Through these actions, it effectively reduces oxidative stress and protects tissue and organ function in various pathological conditions, including nephrotoxicity, ischemia-reperfusion injury, and brain injury.

#### 4.2.3. Daidzein Regulates Antioxidant Signaling Pathways

The core antioxidant mechanism of daidzein resides in the establishment of a multi-level antioxidant regulatory network that operates from gene expression to organelle function by modulating cellular signaling pathways, Specifically, it activates the Nrf2/ARE pathway and enhances its activity at the upstream kinase level, while also exerting a protective effect at the mitochondrial level.

Firstly, daidzein has been reported to activate the Nrf2 pathway, leading to Nrf2 dissociation from Kelch-like ECH-associated protein 1 (Keap1) and nuclear translocation, thereby initiating the expression of antioxidant enzymes such as HO-1 and SOD. In a glycerol-induced AKI rat model, daidzein activates the Nrf2/HO-1 pathway in the kidney and dose-dependently increases the SOD, CAT, and GSH levels. Molecular docking has suggested a potential interaction between daidzein and the Keap1-Nrf2 complex [69]. However, direct evidence such as cysteine modification assays or mutagenesis studies is still needed to verify whether daidzein directly modifies Keap1 cysteine residues. Additionally, in mouse models of traumatic brain injury, daidzein upregulates Nrf2 and HO-1 expression in brain tissue, downregulates Keap1, and inhibits neuroinflammation and apoptosis [70]. Dietary supplementation with daidzein in pregnant sows also activates the ovarian Nrf2/HO-1 pathway, reduces oxidative stress and inflammatory cytokines, and improves reproductive performance [57]. Together, these studies support that the Nrf2/ARE pathway is a key mediator of the sustained antioxidant effects of daidzein, although the exact molecular details of how daidzein interacts with Keap1 remain to be clarified.

Secondly, daidzein enhances Nrf2 activity by regulating upstream kinases such as phosphatidylinositol 3-kinase/protein kinase B (PI3K/Akt) and MAPK. In LPS-induced intestinal epithelial barrier injury models, daidzein inhibits NF-κB signaling by suppressing excessive activation of the PI3K/Akt and p38 signaling pathways, thereby reducing TNF-α and IL-6 release and maintaining the expression of tight junction proteins (ZO-1, occludin, and claudin-1) as well as intestinal epithelial barrier function [73]. In ovarian cancer cells, daidzein induces cell cycle arrest and apoptosis by inhibiting the focal adhesion kinase (FAK) and PI3K/Akt/GSK pathways [74].

In addition, daidzein directly acts on mitochondria, stabilizing membrane potential, reducing ROS leakage, and upregulating SOD2 expression. In human breast cancer (MCF-7) cells, daidzein-induced mitochondrial membrane potential decline and apoptosis are significantly inhibited by the antioxidant N-acetylcysteine (NAC), confirming that daidzein influences cell fate by regulating the mitochondrial redox state [63]. Furthermore, in human liver cancer BEL-7402 cells, daidzein was shown to trigger endogenous apoptosis by increasing ROS levels and reducing mitochondrial membrane potential, further confirming its regulatory effect on mitochondrial function [65].

In summary, daidzein establishes a multi-level, multi-target antioxidant signaling network by activating the core Nrf2/ARE pathway, regulating upstream kinases such as PI3K/Akt and MAPK, and maintaining mitochondrial homeostasis.

## 5. The Role of Daidzein in Diseases via Regulating Inflammation and Oxidative Stress

**Figure 4:**
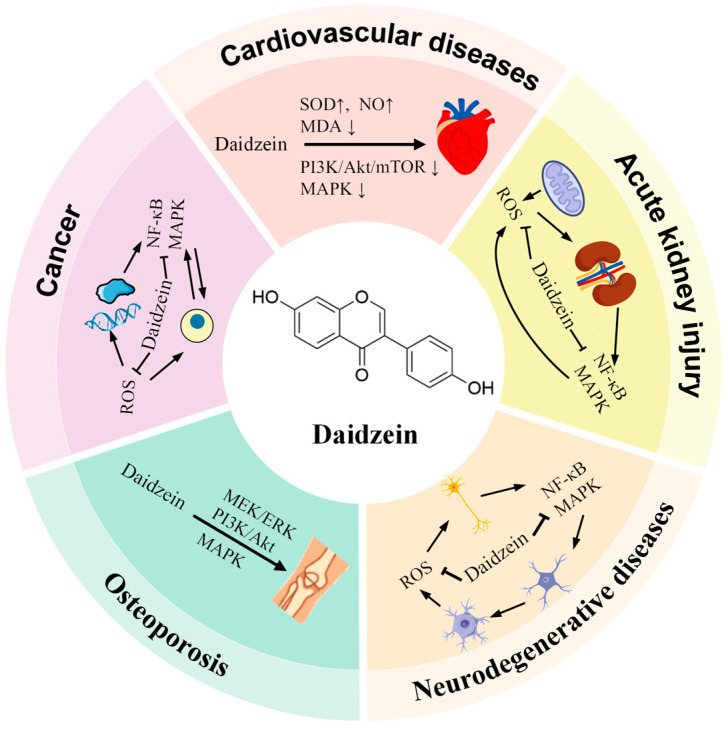
Mechanisms of daidzein against diseases via anti-inflammation and antioxidation. Note: Akt, protein kinase B; ERK, extracellular signal-regulated kinase; MAPK, mitogen-activated protein kinase; MEK, mitogen-activated protein kinase kinase; MDA, malondialdehyde; mTOR, mechanistic target of rapamycin kinase; NF-κB, nuclear factor kappa-B; NO, nitric oxide; PI3K, phosphatidylinositol 3-kinase; ROS, reactive oxygen species; SOD, superoxide dismutase.

Daidzein, as a multi-target active phytoestrogen, exhibits broad intervention potential in the pathological processes of various diseases by modulating inflammation, oxidative stress, and related cellular signaling pathways. Its mechanism of action involves three aspects: fine regulation of specific cellular functions, inhibition or activation of core signaling pathways, and systematic regulation of downstream effector molecules, collectively contributing to protective or beneficial outcomes in different disease models. This section elaborates on the biological effects and molecular mechanisms of daidzein in multiple disorders, including cancer, cardiovascular disease (CVD), and kidney injury. The mechanisms underlying the disease-ameliorating effects of daidzein via anti-inflammatory and antioxidant actions are presented in Figure 4. 

### 5.1. Cancer

Oxidative stress and chronic inflammation promote each other, creating a vicious cycle that drives cancer development. Excessive accumulation of ROS in the body triggers oxidative stress, leading to DNA damage, gene mutations, and oxidative modifications of proteins and lipids. It also activates the NF-κB pathway, promoting the release of inflammatory factors and sustaining a chronic inflammatory state [75]. Immune cells within the inflammatory microenvironment further produce high levels of ROS and inflammatory mediators, thereby exacerbating oxidative damage and inflammatory responses. Consequently, this promotes tumor cell proliferation, anti-apoptotic effects, angiogenesis, invasion, and metastasis [76]. Daidzein exerts anti-cancer effects by inhibiting tumor growth, invasion, and metastasis, and by inducing cancer cell apoptosis via antioxidation, anti-inflammatory mechanisms, and the modulation of multiple signaling pathways.

Daidzein exhibits both chemopreventive and direct antitumor effects, which should be distinguished as separate biological claims. In terms of chemoprevention, animal studies have demonstrated that it elevates the activities of antioxidant enzymes, including SOD and CAT, in liver and breast tissues, reduces lipid peroxidation and oxidative stress, and decreases the incidence of chemically-induced breast tumors by 10% and tumor multiplicity by 37.4%, thereby exerting early anticancer effects [77]. However, these results are from rodent models with small sample sizes, and the doses used are much higher than human dietary exposure, limiting direct translation. Thus, while statistically significant, this chemopreventive efficacy should not be equated with protection against human breast carcinogenesis through dietary daidzein consumption.

At the therapeutic level, daidzein inhibits the progression of various tumors via multiple targets and pathways, with caspase-3/8/9 activation being the key execution mechanism. In osteosarcoma, daidzein exerts its anti-osteosarcoma effect by directly binding to and inhibiting Src kinase activity, thereby downregulating phosphorylation of the downstream ERK signaling pathway. It induces S-phase arrest and activates caspase-3/9 to trigger mitochondrial intrinsic apoptosis [78]. In prostate cancer, daidzein inhibits the NF-κB pathway, enhances the activities of antioxidant enzymes including SOD and GPx, and downregulates the expression of genes such as vascular endothelial growth factor (VEGF) and MMP-2/9. Through these effects, it modulates cell cycle progression, induces cell death via extrinsic apoptosis (caspase-3/8 activation), suppresses angiogenesis and metastasis, and exerts antioxidant effects, indicating its considerable potential in the prevention and adjuvant treatment of prostate cancer [79]. In oral squamous cell carcinoma, daidzein inhibits the MMP2/9-MAPK-EMT axis to reduce invasiveness and induces caspase-3-dependent apoptosis; however, its combination with cisplatin is dose-dependent and requires an appropriate daidzein-to-cisplatin ratio [55]. Of note, the effective concentrations applied in these cell-line and xenograft models are often orders of magnitude above the physiologically attainable plasma levels via diet, meaning that these pro-apoptotic and cell-cycle modulating effects represent pharmacological, not nutritional, actions.

Beyond direct cytotoxicity, daidzein has a clear anti-metastatic effect that is mechanistically distinct from its pro-apoptotic action. In prostate and oral squamous cell carcinomas, daidzein reduces cell migration and invasion by downregulating MMP-2/9, inhibiting MAPK signaling (ERK1/2 and p38 phosphorylation), and reversing epithelial–mesenchymal transition (EMT) [55,79]. Although anti-metastatic activity itself does not directly rely on caspase-mediated apoptosis, metastatic cells ultimately need to be eliminated by apoptosis, and daidzein may synergistically enhance caspase activity in this process.

In addition, daidzein shows strong potential for combination therapy, where activation of caspase-3/8/9 is a core mechanism. When combined with anti-estrogenic drugs, it targets and induces mitochondrial apoptosis in breast cancer cells, inhibits the PI3K/Akt/mTOR pathway, and promotes the expression of the pro-apoptotic protein Bcl-2-associated X protein (Bax) while inhibiting the anti-apoptotic protein B-cell lymphoma-extra large (Bcl-Xl), and significantly activates caspase-3/7/9, thereby exerting synergistic anti-cancer effects [80]. In combination with gefitinib, daidzein increases ROS levels in lung cancer cells, activates apoptotic pathways, and inhibits signal transducer and activator of transcription (STAT), protein kinase B (AKT), and ERK signaling downstream of epidermal growth factor receptor (EGFR). These effects ultimately lead to G0/G1 phase cell cycle arrest, induction of endogenous and exogenous apoptosis, enhanced chemosensitivity, reduced toxicity, and improved chemotherapeutic efficacy [81]. A note of caution: when combined with cisplatin in oral squamous cell carcinoma, low-dose cisplatin shows synergistic cytotoxicity (accompanied by caspase-3 activation), whereas high-dose cisplatin may produce antagonism, indicating the need for optimization of the dosing regimen [55]. Importantly, although these synergistic chemosensitizing effects are promising, they have only been validated under supra-pharmacological conditions in vitro.

Although daidzein has shown anticancer potential in various tumor models, the existing evidence mainly comes from small-sample rodent or in vitro studies, with dosages far exceeding the dietary exposure levels in humans, thus lacking physiological relevance. As a phytoestrogen, its effects may vary depending on tumor type, estrogen receptor status, and microenvironment. Moreover, there is a lack of long-term safety and pharmacokinetic data. Future studies need to be conducted using doses and models that are closer to those in humans for validation.

### 5.2. Acute Kidney Injury

AKI is a clinical syndrome caused by multiple factors and characterized by a rapid decline in renal function within a short period of time. Its core features include a sharp decrease in glomerular filtration rate, retention of nitrogen-containing metabolites, and disruption of water, electrolytes, and acid–base balance [82]. Its pathogenesis is associated with oxidative stress. Extensive accumulation of ROS triggers oxidative stress, directly damaging renal tissue and activating inflammatory pathways. In turn, inflammation-derived factors further exacerbate oxidative stress, forming a vicious cycle that continuously aggravates tubular damage and promotes the rapid deterioration of renal function [83].

Daidzein, with its antioxidant and anti-inflammatory capabilities, breaks the vicious cycle of oxidative stress–inflammation–apoptosis/fibrosis. In a variety of experimental models of kidney injury, daidzein has demonstrated clear protective effects and consistent pathways of action. In a glycerol-induced AKI rat model, pretreatment with daidzein at doses of 25–100 mg/kg given orally for two weeks before glycerol injection significantly improved renal function by reducing the levels of urea, creatinine, and kidney injury molecule 1 (KIM-1); enhances antioxidant capacity by strengthening the Nrf2/HO-1 pathway; and inhibits the NF-κB pathway and inflammatory factors such as IL-1β and TNF-α. Furthermore, while regulating apoptosis-related proteins (reducing Bax/caspase-3 and increasing Bcl-2), it alleviates renal histopathological damage through multiple pathways [69]. In gentamicin-induced nephrotoxicity models (MDCK cells and adult zebrafish), daidzein at concentrations of 25–100 μM exerts a dose-dependent protective effect by increasing SOD and GSH activity, reducing the production of lipid peroxides, NO, and ROS to suppress oxidative stress, and downregulating the expression of cytokines such as COX-2, TNF-α, and IL-1β to reduce inflammation, thereby reversing renal pathological damage [84].

Although kidney damage can be alleviated through dietary adjustments and lifestyle improvements, the problem of cisplatin-induced nephrotoxicity is still difficult to avoid [51]. Multiple studies have confirmed that daidzein has protective effects against cisplatin chemotherapy-induced damage. In one study, daidzein, administered at a pharmacological dose of 200 mg/kg body weight 1 h after cisplatin injection, significantly improved the renal function indicators, attenuated oxidative stress and inflammatory responses in renal tissue, and inhibited tubular apoptosis [66]. Furthermore, it effectively ameliorated cisplatin-induced abnormalities in hematological parameters, enhanced hepatic antioxidant capacity, and reduced liver tissue injury. Collectively, these protective effects are mainly attributed to its multifaceted mechanisms, including anti-oxidation, anti-inflammation, and anti-apoptosis, acting in a dose-dependent manner [85]. However, a critical concern is whether daidzein could compromise the antitumor efficacy of cisplatin. Recent evidence in oral squamous cell carcinoma revealed a dose-dependent interaction: daidzein synergizes with low-dose cisplatin yet antagonizes high-dose cisplatin by protecting tumor cells via its antioxidant activity [55]. This biphasic effect implies that daidzein might inadvertently protect malignant cells under specific contexts, suggesting that its application as a protective adjuvant warrants further validation in tumor-bearing models, alongside the careful optimization of dosing schedules.

The current evidence mainly comes from preclinical studies and requires clinical validation. Most of the existing experiments administer daidzein preventively before the injury occurs, while there is insufficient research on therapeutic administration. Furthermore, the long-term safety of daidzein has not been evaluated, nor has its potential impact on the anti-tumor efficacy of cisplatin been assessed.

### 5.3. Cardiovascular Diseases

CVD is one of the leading causes of death, and its onset is closely related to vascular endothelial dysfunction, myocardial injury, and thrombosis [86]. Daidzein exerts multilevel cardiovascular protection by improving vascular reactivity, protecting cardiomyocytes, and inhibiting platelet activation.

Vascular endothelial injury caused by diabetes is an early key pathological basis for the occurrence and development of CVD [87]. Studies have shown that in streptozotocin-induced diabetic rat models, daily intragastric administration of daidzein for 7 weeks enhanced the NO and prostaglandin pathways, reduced MDA content in aortic tissue, and increased SOD activity. It also significantly enhanced the endothelium-dependent vasodilation response of the aortic ring to acetylcholine and reduced the excessive contraction response of the vessel to phenylephrine, thereby alleviating vascular endothelial injury and pre-atherosclerotic lesions induced by metabolic diseases such as diabetes [88].

In doxorubicin-induced cardiotoxicity models, daidzein pretreatment improves cardiac function decline, reduces pathological damage such as myocardial fibrosis disorder, apoptosis, and over-activation of autophagy, and prevents progression to heart failure and cardiomyopathy. It acts by inhibiting the PI3K/Akt/mTOR pathway, downregulating phosphorylated Akt, cleaved caspase-3, and LC3-II while upregulating Bcl2 and cyclin D1 [89]. In terms of antithrombotic effects, daidzein inhibits collagen-induced platelet activation, granule release, and clot contraction; reduces thromboxane A2 (TXA_2_) production by lowering cytosolic phospholipase A2 (cPLA_2_) phosphorylation; and increases cyclic adenosine monophosphate (cAMP). It inhibits platelet aggregation by modulating the PI3K/Akt/GSK3α/β and MAPK signaling pathways and exerts synergistic effects with low-dose aspirin. These actions provide promising preventive and therapeutic strategies for ischemic cardiovascular diseases including atherosclerosis, coronary heart disease, and cerebral infarction [90].

These preclinical results suggest that daidzein can alleviate vascular lesions in metabolic diseases, protect the myocardium from drug damage, and intervene in the process of thrombosis, providing a new natural compound option for further investigation in the prevention and treatment of cardiovascular diseases.

### 5.4. Neurodegenerative Disease

Neurodegeneration refers to a class of pathological processes in which neurons undergo progressive and irreversible damage and death, resulting in the gradual decline of corresponding functions. It is the core pathological feature of many neurological disorders such as Parkinson’s disease (PD), Alzheimer’s disease (AD), and amyotrophic lateral sclerosis [91]. The core mechanism involves protein misfolding and aggregation, accompanied by mitochondrial dysfunction, neuroinflammation, oxidative stress, and other cellular cascade reactions, ultimately resulting in neuronal energy depletion and death [92].

PD, as a common neurodegenerative disease, is pathologically characterized by loss of dopaminergic neurons in the substantia nigra and aggregation of α-synuclein, presenting with motor and non-motor symptoms [93]. Oxidative stress and neuroinflammation are the core mechanisms, and they promote each other to form a vicious cycle. Abnormalities in mitochondrial and dopamine metabolism produce a large amount of ROS, damaging neurons and promoting abnormal aggregation of α-synuclein. Aggregated proteins activate microglia to release pro-inflammatory factors such as TNF-α and IL-1β, further damaging neurons and intensifying oxidative stress, thereby promoting disease progression [94]. 1-Methyl-4-phenyl-1,2,3,6-tetrahydropyridine (MPTP), a commonly used neurotoxin for inducing Parkinson’s disease models in mice, is widely used in Parkinson’s disease research. In an in vivo MPTP-induced C57BL/6 mouse PD model and an in vitro LPS-induced BV2 microglial cell model, treatment with daidzein at 10, 25, and 50 µM in vitro or orally at 50 or 75 mg/kg for 30 days in vivo improved motor function, restored dopamine levels, reduced inflammation, and corrected abnormal brain tissue structure in PD mice. In vitro experiments showed that daidzein was non-cytotoxic and dose-dependently reduced levels of NO, ROS, PGE2, IL-6, and IL-1β [46].

AD is a neurodegenerative disease centered on progressive cognitive decline. Its typical pathology is the deposition of beta-amyloid (Aβ) plaques in the brain, and hyperphosphorylation of tau protein to form neurofibrillary tangles, ultimately leading to massive neuronal death [95]. Metal elements such as iron, zinc, and copper promote Aβ aggregation, and Aβ significantly increases ROS production, seriously affecting neuronal metabolism [96]. In an AD-related Aβ oligomer injury model, daidzein at 0.5 µM with 2-h pretreatment significantly improved Aβ-induced decline in cell viability, reduced ROS and MDA production, maintained mitochondrial membrane potential, inhibited neuroinflammation and MAPK pathways, and downregulated COX-2, IL-1β, NF-κB, and their phosphorylation levels. It also alleviated neuronal and glial cell damage, suggesting a potential therapeutic approach for Aβ-related neurodegenerative diseases in vitro such as Alzheimer’s disease [97].

These experiments indicate that research on the neuroprotective effects of daidzein mediated through anti-inflammatory and antioxidant mechanisms is still at an early stage. Further rigorous in vivo verification, long-term safety assessment, and mechanism analysis are required before considering its clinical application.

### 5.5. Type 2 Diabetes Mellitus

Type 2 diabetes mellitus is a chronic metabolic disorder characterized mainly by insulin resistance and progressive decline of pancreatic β-cell function, often accompanied by chronic low-grade inflammation, oxidative stress, dyslipidemia, and mitochondrial dysfunction [98]. Given the significantly higher fracture risk in diabetic patients, elucidating how diabetic milieu affects bone is clinically crucial. In this regard, high glucose-induced oxidative stress, advanced glycation end products, and chronic inflammation interfere with key pathways, including receptor activator of NF-κB ligand/osteoprotegerin (RANKL/OPG), insulin signaling, and peroxisome proliferator-activated receptor γ (PPARγ). This interference shifts the balance toward osteoclastic resorption at the expense of osteoblastic formation, thereby directly linking diabetic dysregulation to skeletal impairment [99]. Among the various natural products, daidzein—a major isoflavone derived from soybeans—has garnered considerable attention for its potential in type 2 diabetes intervention due to its pleiotropic regulation of glucose and lipid metabolism, as well as its anti-inflammatory and antioxidant properties [100].

Researchers used a streptozotocin-induced diabetic rat model and administered 25, 50, and 100 mg/kg daidzein orally for 4 consecutive weeks, finding that it significantly inhibited the expression of nicotinamide adenine dinucleotide phosphate (NADPH) oxidase subtype NADPH oxidase 4 (NOX-4) in the myocardium of diabetic rats [101]. Similar results were observed in the sciatic nerve tissue, where the same dose of daidzein significantly inhibited NOX-4 expression, with an effect comparable to that of the positive control drug pregabalin at 30 mg/kg [102]. Daidzein reduced the generation of superoxide radicals, increased the activity of endogenous antioxidant enzymes, and lowered the level of the lipid peroxidation product malondialdehyde, thereby effectively alleviating oxidative stress damage caused by high glucose.

Regarding anti-inflammatory effects, in an obese mouse model induced by a high-fat and high-sucrose diet, feeding mice with 0.1% daidzein for 12 weeks reduced lymphocyte infiltration, fibrosis, and abnormal vascular proliferation in cardiac tissue. It also decreased TNF-α, IL-6, and monocyte chemoattractant protein-1 (MCP-1) levels in adipose tissue and serum while upregulating the anti-inflammatory factor adiponectin [103]. However, this study used only one dose and did not assess long-term safety.

In cell experiments, daidzein at concentrations of 6.25 to 25 μM, commonly 25 μM, activated peroxisome proliferator-activated receptor α (PPARα) and PPARγ, inhibited JNK phosphorylation, and thereby downregulated the transcription of pro-inflammatory factors [104]. Additionally, daidzein directly inhibited PARP-1 enzyme activity, reduced the PARylation modification of the NF-κB subunit RelA/p65, and blocked NF-κB-mediated expression of pro-inflammatory chemokines [60]. However, in this mechanistic study, the in vitro concentration was 10 μmol/L, which is much higher than the physiological concentrations in humans, and the study focused on only a single chemokine.

In conclusion, daidzein exhibits clear protective effects against type 2 diabetes, mediated by its anti-inflammatory and antioxidant properties. However, common limitations include short experimental duration, high doses of daidzein, and a lack of clinical validation. Given that diabetes adversely impacts bone turnover, evaluating daidzein’s skeletal effects in addition to its metabolic regulation is warranted. The following subsection thus details its mechanisms in osteoporosis.

### 5.6. Osteoporosis

Osteoporosis is characterized by an imbalance between osteoclast bone resorption and osteoblast bone formation, reduced bone mass, destruction of bone microstructure, and an increased risk of fractures [105]. Key pathogenic mechanisms include estrogen deficiency, oxidative stress, inflammatory response, cellular senescence, and abnormal epigenetic regulation [106]. ROS and pro-inflammatory factors promote osteoclast generation and inhibit osteoblast function through pathways such as MAPK, and miRNAs have reciprocal regulatory effects with oxidative stress and inflammation [107].

Daidzein, as a selective estrogen receptor modulator, mimics the beneficial effects of estrogen on bone metabolism by promoting both bone formation and inhibiting excessive bone resorption. In human osteogenic MG-63 cells, daidzein treatment at concentrations ranging from 0.01 to 10 μmol/L with an optimal effect at 0.1 μmol/L promotes cell proliferation, enhances alkaline phosphatase activity, increases type I collagen secretion and mineralized nodule formation, and inhibits cisplatin-induced apoptosis. These effects promote osteoblast proliferation, differentiation, and the inhibition of apoptosis through ER-dependent MEK/ERK, PI3K/Akt pathways [108]. In terms of inhibiting bone resorption and regulating bone metabolism, daidzein upregulates bone sialoprotein (BSP) mRNA expression via the nuclear factor Y (NF-Y) transcription factor and specific sequences on the BSP promoter. The study suggests that daidzein can promote bone formation and inhibit tumor bone metastasis through specific transcriptional regulatory mechanisms [109].

### 5.7. Other Diseases

Daidzein also exerts beneficial effects against multiple other diseases. It activates the ERK1/2 pathway to reduce oxidative stress and promote neural plasticity, thereby producing antidepressant and neuroprotective effects [110]. Daidzein also downregulates transient receptor potential vanilloid 1 (TRPV1) and P2Y receptors, activates the Nrf2/HO-1 pathway, inhibits pain signaling, displays antioxidant and anti-inflammatory activities, and repairs nerve injury [111]. Moreover, it suppresses inflammatory factors including TNF-α and IL-6, downregulates atrophy-related genes and proteins such as Fbxo32, Trim63, Foxo1, and MuRF1, and ultimately improves obesity, glycolipid metabolism, and muscle atrophy [112]. The anti-inflammatory and antioxidant potential of daidzein in diseases has also been widely documented, as summarized in Table 2.

## 6. Pharmacokinetic Limitations and Examples of Nanoformulation-Based Delivery Systems

### 6.1. Pharmacokinetic Limitations of Daidzein

Daidzein, a dietary isoflavone mainly derived from soybeans and soy products, exhibits multiple biological activities, including antioxidant, anti-inflammatory, antibacterial, and anti-cancer effects. However, its limited oral bioavailability significantly restricts its application potential. This limitation stems from its complex absorption and metabolism characteristics. In its natural state, daidzein exists mostly as poorly water-soluble glycosides and requires hydrolysis by intestinal mucosal β-glucosidase or intestinal flora to be absorbed via passive diffusion [113]. Following absorption, the compound rapidly undergoes phase II metabolism in the liver, including glucuronidation and sulfation [114], resulting in more water-soluble conjugates (75% glucuronic acid conjugates and 24% sulfate conjugates in plasma), while less than 1% of free aglycones enter the colon. This implies that the biological effects of daidzein may primarily depend on its circulating metabolites rather than the parent compound itself.

Approximately 90% of the components are not absorbed by the small intestine and need to be further converted by the microbiota into metabolites such as dihydrodaidzein, O-demethylangolensin, and equol [115]. Among these, equol has attracted particular attention due to its high estrogen receptor affinity and antioxidant activity. The production of equol shows significant individual differences, with Asian populations generally having a higher proportion of producers than Western populations [116]. Specifically, equol production depends on whether the gut microbiota contains specific strains capable of converting daidzein to equol [117], and globally, only a portion of the population possess this ability [118].

Dietary patterns, soybean processing methods, and the composition of the gut microbiota all further affect its absorption efficiency. For example, co-ingestion of glucose or other glycoside substrates may competitively inhibit β-glucosidase activity, reducing the release rate of aglycones. Researchers have found that when consuming soy milk containing glucoside conjugates, daidzein absorption is faster than from solid soy foods, with a difference of up to 2 h [119]. Another study reported that insoluble fiber, such as inulin, may increase daidzein absorption [120]. Furthermore, sex and hormonal status play an important regulatory role in daidzein metabolism. Estrogen levels can affect phase II metabolic enzyme activity and gut microbiota composition. Premenopausal women or individuals receiving estrogen replacement therapy tend to have higher equol-producing capacity and total isoflavone absorption rates than men or postmenopausal women [121].

### 6.2. Examples of Nanoformulation-Based Delivery Systems

Recently, nanodelivery systems have become a promising approach to surmount the inherent limitations of daidzein. A variety of nanocarriers have been developed for daidzein delivery, including polymeric nanoparticles [122], nanosuspensions [123], and solid lipid nanoparticles [124]. These formulations markedly improve daidzein solubility, stability, and freeze-drying redispersibility while achieving sustained release in the gastrointestinal tract and targeted delivery to lesion sites in preclinical settings [15]. The engineered daidzein-loaded nanocarriers have demonstrated potent antioxidant and anti-inflammatory properties in vitro and in animal models. They have also been shown to combat drug-resistant bacterial infections [125], exert anti-tumor effects [14], and alleviate osteoporosis [126] in these models. This section focuses on the dual antioxidant and anti-inflammatory actions of nanodelivered daidzein and reviews recent progress in nanotechnology-based bioavailability enhancement. However, improved solubility does not necessarily translate into better systemic exposure or in vivo bioavailability, because most studies only report enhanced in vitro activity and provide limited direct in vivo evidence. Further investigation is required to confirm whether solubility enhancement genuinely improves systemic exposure.

In terms of improving basic physicochemical properties, some researchers have prepared inclusion complexes of daidzein with poly(amidoamine) (PAMAM) and poly(propylene imine) (PPI) dendrimers, which increased the solubility of daidzein by 186-fold and 650-fold, respectively, at a concentration of 0.36 mM. Among them, the PAMAM inclusion complex showed better stability (94% recovery rate after 30 days) and lower cytotoxicity while maintaining its antioxidant activity [127]. Other researchers used an ultrasonic-assisted anti-solvent precipitation method to construct corn zein nanoparticles with carrageenan and sodium alginate as double-layer stabilizers. Under optimal conditions, the encapsulation efficiency reached 90.36%, significantly improving the stability and freeze-dried redispersibility of daidzein under different pH and ionic strength conditions, achieving sustained release in the gastrointestinal tract, and markedly enhancing DPPH and ABTS radical scavenging capacity [128].

Researchers developed daidzein-loaded nanoliposomes with a particle size of 293.0 ± 46.5 nm, which showed dose-dependent improvement of symptoms in a diabetic mouse model. These nanoliposomes functioned by enhancing antioxidant enzyme activity, inhibiting the expression of inflammation-related genes, and regulating the expression of glucose transporters. At a dose of 100 mg/kg, the effect was comparable to that of metformin, and no obvious toxic side effects were observed [129]. Another research team synthesized daidzein-modified gold nanoparticles through a one-pot green method, with an average particle size of 25.78 nm. The minimum inhibitory concentration against 15 clinical carbapenem-resistant Enterobacteriaceae strains was 8–16 μg/mL. These nanoparticles exerted a synergistic antibacterial effect through multiple mechanisms, including disruption of cell membranes, induction of ROS, and interference with metabolism. In vivo administration achieved a 72-h survival rate of 100% in infected mice, and the nanoparticles showed good biocompatibility [130].

Additionally, scholars developed chitosan-chondroitin sulfate-daidzein nanocomplexes, which cumulatively released 79.66% of daidzein within 96 h under physiological conditions. They effectively improved glucocorticoid-induced osteoporosis by downregulating ROS levels and regulating the expression of bone metabolism-related genes [131]. Furthermore, another study constructed solid lipid nanoparticles loaded with irinotecan and daidzein, which were surface-modified with hyaluronic acid and bovine serum albumin and coated with chitosan. With a particle size of 215.6 nm and an encapsulation efficiency of 82%, these nanoparticles targeted colon cancer cells via receptor-mediated endocytosis, induced cell cycle arrest and apoptosis, and achieved a synergistic anticancer effect [132].

In summary, nanocarrier technology overcomes the poor water solubility, low stability, and low bioavailability of daidzein via particle size regulation, crystal transformation, intermolecular interactions, and targeted modification, thereby improving its bioavailability. However, as stated above, solubility improvement alone does not guarantee enhanced systemic exposure. Rigorous pharmacokinetic studies are required to confirm in vivo bioavailability gains. Various nanoformulations are tailored to different applications, offering a reference for transforming daidzein into clinical formulations and functional foods, and for developing other hydrophobic bioactive compounds. An overview of its nano-based delivery systems is presented in Table 3.

## 7. Conclusions and Prospects

In summary, daidzein is a natural isoflavone plant estrogen with anti-inflammatory and antioxidant activities. By synergistically regulating signaling pathways such as NF-κB/MAPK and Nrf2/HO-1, it demonstrates therapeutic potential in various models including cancer, cardiovascular diseases, kidney damage, and bone diseases. However, its poor water solubility, low oral bioavailability, rapid phase II metabolism in the liver, and individual differences in intestinal flora metabolism severely restrict its clinical translation.

Existing evidence indicates that nanodelivery systems can significantly enhance the solubility, stability, and targeting ability of daidzein. Various disease models have confirmed that nanoformulations can significantly improve therapeutic efficacy, effectively overcoming the pharmacokinetic bottleneck of the drug and providing a feasible path for the development of clinical formulations and functional foods. However, current research still has key limitations: the majority of evidence comes from preclinical models, lacking clinical research support; there are significant individual differences in in vivo metabolism, affected by intestinal flora and phase II metabolic enzymes; the long-term safety of nanoformulations has not been systematically evaluated; and there is a lack of standardized characterization, pharmacokinetic, and toxicological protocols, which limits data comparability.

Oxidative stress and inflammation are a common pathological basis of various diseases. Daidzein has broad prospects in the prevention and treatment of related diseases. Future research should prioritize the comparison of different nanocarriers under standardized conditions, develop active targeting preparations, establish scalable and compliant processes, and conduct in-depth toxicological, pharmacokinetic, and exploratory clinical trials to promote its development from a natural component to a clinical drug and functional food.

## Figures and Tables

**Table 1:** Main plant sources of daidzein.

Family	Genus	Plant	Medicinal Part	Dry Weight (mg·kg^−1^)	Reported Bioactivities of the Plant	Ref.
Fabaceae	Glycine	*Glycine max* (L.) Merr.	Seeds	1.7–23.5	Antioxidant, anti-tumor, anti-diabetic, anti-obesity, anti-inflammatory, cardio and neuroprotective activities	[34]
	Trifolium	*Trifolium pratense* L.	Leaves	720–1910	Relieve menopausal symptoms, prevent osteoporosis and cardiovascular diseases	[35]
	Medicago	*Medicago sativa* L.	Flower	<2300	Antioxidant, antibacterial, anti-inflammatory, neuroprotective	[36]
	Pueraria	*Pueraria lobata*	Root	251.7–355.2	Antioxidant, anti-inflammatory, anti-tumor, neuroprotective and cardiovascular protective	[37]
	Pisum	*Pisum sativum* L.	Seeds	1.7–2.7	Anti-tumor, antibacterial, anti-inflammatory, and antioxidant	[38]
	Vicia	*Vicia faba* L.	Seeds	5.0	Anti-tumor, antioxidant, lowering blood pressure, lowering blood sugar	[28]
	Phaseolus	*Phaseolus vulgaris* L.	Root	340–1020	Anti-tumor, anti-inflammatory, antioxidant	[39]
	Psoralea	*Psoralea corylifolia* L.	Seeds	61.2–134.3	Antibacterial, antiviral, anti-inflammatory, antioxidant, anti-tumor, anti-osteoporosis, neuroprotective	[40]
	Butea	*Butea superba Roxb.*	Root	N/A	Enhance the developmental maturation of reproductive organs, antioxidant	[41]
Iridaceae	Iris	*Iris pseudacorus* L.	Root	N/A	Antioxidant, anti-inflammatory, and anti-tumor	[42]
Anacardiaceae	Pistacia	*Pistacia vera* L.	Nut kernel	37	Cardioprotective and anti-tumor	[31]
Amaranthaceae	Chenopodium	*Chenopodium quinoa* Willd.	Seeds	11.5	Breast cancer, cardiovascular diseases	[32]

Note: N/A, not applicable.

**Table 2:** Mechanisms of daidzein in related diseases via anti-inflammatory and antioxidant effects.

Disease		Model	Study Design	Dose	Duration	Route	Mechanism	Ref.
Cancer	Osteosarcoma	BALB/c nude mice	In vivo	20 mg/kg	9 d	i.v.	↓Src, ↓Phosphorylation of ERK	[78]
		143B and U2OS osteosarcoma cells	In vitro	0–500 µM	72 h	N/A		
	Oral squamous cell carcinoma	Ca9-22, SAS, SG cell lines	In vitro	25–200 µM	24 h	N/A	↓MMP-2/9, ↓Phosphorylation of ERK1/2 and p38	[55]
	Breast cancer	MCF-7, and MDA MB-231 cells	In vitro	10–200 µM	48 h	N/A	↓PI3K/Akt/mTOR pathway, ↓Phosphorylation of Akt and Mtor, ↑Bax, ↓Bcl-xL	[80]
	Lung adenocarcinoma	A549, BEAS-2B, H9C2, Clone 9 cells, H1975 and LoVo cancer cells	In vitro	100–300 µM	24 h	N/A	↑ROS, ↑ASK1/JNK/c-Jun pathway, ↓STAT, ↓AKT, ↓ERK	[81]
AKI	Acute kidney injury	Wistar albino rats	In vivo	25–100 mg/kg	14 d	i.g.	↑SOD, ↑CAT, ↑GSH, ↓IL-1β, ↓TNF-α, ↓NF-κB, ↓MPO, ↑Nrf2/HO-1 pathway	[69]
	Renal toxicity	Zebrafish	In vivo	25–100 µM	72 h	i.p.	↑SOD, ↑GSH, ↓LPO, ↓NO, ↓ROS, ↓COX-2, ↓TNF-α, ↓IL-1β	[84]
		MDCK cells	In vitro	25–100 µM	24 h	N/A		
	Kidney injury	Swiss albino rats	In vivo	25–100 mg/kg	10 d	i.p.	↓MDA, ↑GSH, ↑SOD, ↓TNF-α, ↓IL-6 ↓MAPK pathway	[51]
	Kidney injury	C57BL/6 mice	In vivo	200 mg/kg	2 d	i.p.	↓ROS, ↓NOX2, ↑GSH, ↑SOD, ↑GPx, ↓TNF-α, ↓IL-18, ↓MCP-1,	[66]
CVD	Diabetes	Albino Wistar rats	In vivo	5, 10 mg/kg	49 d	i.g.	↑NO, ↓MDA, ↑SOD	[88]
	Cardiac injury	SD rats	In vivo	20, 40 mg/kg	42 d	s.c.	↓PI3K/Akt/mTOR and PI3K/Akt pathway, ↓Phosphorylation of Akt	[89]
	Thrombus	Platelet	In vitro	12.5–50 µM	2 min	N/A	↑cAMP, ↓PI3K/Akt/GSK3αβ and MAPK pathway	[90]
Neurodegenerative diseases	PD	C57BL/6 mice	In vivo	50, 75 mg/kg	30 d	i.g.	↓NO, ↓ROS, ↓PGE2, ↓IL-6, ↓IL-1β	[46]
		BV2 microglial cells	In vitro	10–50 µM	12 h	N/A	↓ROS, ↓MDA, ↓COX2, ↓IL-1β, ↓NF-κB, ↓NO, ↓MAPK pathway	
	AD	SH-SY5Y and C6 cells	In vitro	0.5 µM	2 h	N/A		[97]
Type 2 diabetes mellitus	Diabetes	SD rats	In vivo	25–100 mg/kg	28 d	i.g.	↓NOX-4, ↓MDA, ↑GSH, ↑SOD, ↑CAT, ↑AMPK	[101]
	Diabetes	SD rats	In vivo	25–100 mg/kg	28 d	i.g.	↓NOX-4, ↓MDA, ↑GSH, ↑SOD, ↑CAT	[102]
	Diabetes	C57BL/6J mice	In vivo	1000 mg/kg	84 d	p.o.	↑PPARγ, ↓MCP-1, ↓TNF-α	[103]
	Diabetes	3T3 L1 adipocytes + RAW264 macrophages	In vitro	25 µM	24 h	N/A	↓PPARα/γ, p-JNK	[104]
	Diabetes	MLE-12 mouse lung epithelial cells	In vitro	10 µM	6 h	N/A	↓PARP-1, ↓RelA/p65 PARylation, ↓NF-κB, ↓Cxcl2	[60]
Osteoporosis	Postmenopausal osteoporosis	Human osteoblast- like MG-63 cells	In vitro	0.01–10 µM	6 d	N/A	↑MEK/ERK and PI3K/Akt pathway	[108]

Note: i.g., oral gavage; i.p., intraperitoneal injection; i.v., intravenous injection; N/A, not applicable; p.o., oral administration; s.c., subcutaneous injection.

**Table 3:** Nanocarrier formulations for enhancing the solubility and bioavailability of daidzein.

Type	Size (nm)	PDI	Zeta Potential (mV)	EE/DL (%)	Release Curve	Stability	Evidence Level	Toxicity	Key Outcomes	Ref.
Nano drug delivery liquid formulation	N/A	N/A	N/A	Solubility enhancement: 186-fold (PAMAM), 650-fold (PPI)	PAMAM: 6 h release 27.0%, 12 h release 33.4% PPI: 6 h release 9.3%, 12 h release 15.6%	30-d recovery: 94% (PAMAM), 84% (PPI)	In vitro activity only (no PK, no animal study)	PAMAM has no significant toxicity; PPI has high cytotoxicity.	Improved solubility; sustained release; PPI highly cytotoxic, PAMAM safer	[127]
Biopolymer composite nanodelivery formulation	165.7	0.268	<–30 (abs)	90.36%	Simulated GI digestion: 47.6% after 2 h in gastric; 73.8% after 4 h in intestinal	pH 4–8 stable; 4 °C: size 165.7→177.2 nm over 20 d	In vitro activity only (ABTS/DPPH and simulated digestion, no PK)	N/A	Enhanced antioxidant activity and sustained release in simulated GI tract	[128]
Nanoparticle liposome delivery formulation	293.0	0.270	–27.22	Drug loading only: 389 mg/g DW	N/A	Only zeta potential reported, no long-term stability data	In vivo pharmacodynamics, no PK (measured glucose/lipids, no AUC/Cmax)	No obvious damage to liver and pancreas (histopathology)	Reduced blood glucose, improved lipid profile, and antioxidant status in diabetic mice	[129]
Antibacterial green synthesized metal nanomaterials	25.78	0.433	−18.5	N/A (surface-modified, not encapsulated)	N/A	Electrostatic stability	In vivo pharmacodynamics, no PK (survival rate, no drug concentration–time data)	No hemolysis, no cytotoxicity	100% survival in CRE-infected mice; membrane disruption and ROS induction	[130]
Polymer nanoparticle delivery formulation	401.2	0.147	+43.55	Approx. 85.6% (calculated from R%, but EE not explicitly given)	96 h release 79.66%	N/A	In vivo pharmacodynamics, no PK (zebrafish osteoporosis model, no PK)	Low toxicity and good biocompatibility	Reduced oxidative stress, restored bone mineralization, upregulated OPG/downregulated RANKL in zebrafish	[131]
Composite solid lipid nanoparticles	215.6	0.178	–20.5	90% (total for both drugs)	Released 23% in the first 2 h, followed by continuous release; it conforms to the Higuchi model (R^2^ = 0.9743)	6-month stable at 4 °C/25 °C	In vivo pharmacodynamics, no PK (rat colon cancer model, no AUC/Cmax)	Safe and effective	Synergistic anticancer effect (CI = 0.5); restored colon architecture; downregulated CEA and Ki-67	[132]

Note: AUC, area under the curve; CI, combination index; Cmax, maximum plasma concentration; CRE, carbapenem-resistant Enterobacteriaceae; DL, drug loading; DW, dry weight, EE, encapsulation efficiency; OPG, osteoprotegerin; N/A: not applicable; PDI: polydispersity index. PK, pharmacokinetics; RANKL, receptor activator of nuclear factor-κB ligand; ROS, reactive oxygen species.

## Data Availability

No data were used for the research described in the article.

## Abbreviations

The following abbreviations are used in this manuscript:

AD	Alzheimer’s disease
AKI	Acute kidney injury
AKT	Protein kinase B
ASC	Apoptosis-associated speck-like protein containing a CARD
AUC	Area under the curve
Aβ	Beta-amyloid
Bax	Bcl-2-associated X protein
Bcl-xL	β-cell lymphoma-extra large
Bcl-2	β-cell lymphoma 2
BSP	Bone sialoprotein
cAMP	Cyclic adenosine monophosphate
CAT	Catalase
Caspase-1	Cysteine-aspartic acid protease 1
CD40	Cluster of differentiation 40
CD80	Cluster of differentiation 80
CD86	Cluster of differentiation 86
CI	Combination index
Cmax	Maximum plasma concentration;
COX-2	Cyclooxygenase-2
cPLA_2_	Cytosolic phospholipase A2
CRE	Carbapenem-resistant Enterobacteriaceae;
CVD	Cardiovascular disease
CXCL	C-X-C Motif chemokine ligand
DCs	Dendritic cells
DL	Drug loading
DW	Dry weight
EE	Encapsulation efficiency
EGFR	Epidermal growth factor receptor
EMT	Epithelial–mesenchymal transition
ERK1/2	Extracellular signal-regulated kinase 1/2
ERβ	Estrogen receptor β
ETC	Electron transport chain
FAK	Focal adhesion kinase
GPx	Glutathione peroxidase
GSH	Glutathione
HO-1	Heme oxygenase-1
i.g.	Intragastric gavage
IKK	Inhibitor of kappa B kinase
IkBα/β	Inhibitor of kappa B α/β
IL-6	Interleukin-6
IL-8	Interleukin-8
IL-18	Interleukin-18
IL-1β	Interleukin-1β
iNOS	Inducible nitric oxide synthase
i.p.	Intraperitoneal injection
i.v.	Intravenous injection
JNK	c-Jun N-terminal kinase
Keap1	Kelch-like ECH-associated protein 1
KIM-1	Kidney injury molecule 1
LPS	Lipopolysaccharide
MAPK	Mitogen-activated protein kinase
MCP-1	Monocyte chemoattractant protein-1
MDA	Malondialdehyde
MHC class II	Major histocompatibility complex class II
MMP-2	Matrix metallopeptidase 2
MPTP	1-Methyl-4-phenyl-1,2,3,6-tetrahydropyridine
MyD88	Myeloid differentiation primary response 88
N/A	Not applicable
NAC	N-Acetylcysteine
NADPH	Nicotinamide adenine dinucleotide phosphate
NF-Y	Nuclear factor Y
NF-κB	Nuclear factor kappa-B
NF-κB P65	Nuclear factor kappa-light-chain-enhancer of activated B cells p65 subunit
NLRP3	NOD-like receptor thermal protein domain associated protein 3
NO	Nitric oxide
NOX-4	NADPH oxidase 4
NQO1	NAD(P)H dehydrogenase quinone 1
Nrf2	Nuclear factor erythroid 2-related factor 2
OSCC	Oral squamous cell carcinoma
PAMAM	Poly(amidoamine)
PARP-1	Poly(ADP-ribose) polymerase-1
PD	Parkinson’s disease
PDI	Polydispersity index
PGE2	Prostaglandin E2
PI3K/Akt	Phosphatidylinositol 3-kinase/protein kinase B
PK	Pharmacokinetics
p.o.	Oral administration
PPARα	Peroxisome proliferator-activated receptor α
PPARγ	Peroxisome proliferator-activated receptor γ
PPI	Poly(propylene imine)
PRP	Platelet-rich plasma
RANKL/OPG	Receptor activator of NF-κb ligand/osteoprotegerin
ROS	Reactive oxygen species
s.c.	Subcutaneous injection
SOD	Superoxide dismutase
STAT	Signal transducer and activator of transcription
TGF-β1	Transforming growth factor-β
TLR4	Toll-like receptor 4
TNF-α	Tumor necrosis factor-α
TRPV1	Transient receptor potential vanilloid 1
TXA_2_	Thromboxane A2
VEGF	Vascular endothelial growth factor
